# Silence, Solitude, and Serotonin: Neural Mechanisms Linking Hearing Loss and Social Isolation

**DOI:** 10.3390/brainsci10060367

**Published:** 2020-06-12

**Authors:** Sarah M. Keesom, Laura M. Hurley

**Affiliations:** 1 Department of Biology, Utica College, Utica, NY 13502, USA; 2 Center for the Integrative Study of Animal Behavior, Department of Biology, Indiana University, Bloomington, IN 47405, USA; lhurley@indiana.edu

**Keywords:** social isolation, serotonin, hearing loss, communication, auditory, social buffering

## Abstract

For social animals that communicate acoustically, hearing loss and social isolation are factors that independently influence social behavior. In human subjects, hearing loss may also contribute to objective and subjective measures of social isolation. Although the behavioral relationship between hearing loss and social isolation is evident, there is little understanding of their interdependence at the level of neural systems. Separate lines of research have shown that social isolation and hearing loss independently target the serotonergic system in the rodent brain. These two factors affect both presynaptic and postsynaptic measures of serotonergic anatomy and function, highlighting the sensitivity of serotonergic pathways to both types of insult. The effects of deficits in both acoustic and social inputs are seen not only within the auditory system, but also in other brain regions, suggesting relatively extensive effects of these deficits on serotonergic regulatory systems. Serotonin plays a much-studied role in depression and anxiety, and may also influence several aspects of auditory cognition, including auditory attention and understanding speech in challenging listening conditions. These commonalities suggest that serotonergic pathways are worthy of further exploration as potential intervening mechanisms between the related conditions of hearing loss and social isolation, and the affective and cognitive dysfunctions that follow.

## 1. Social Isolation Can Occur with Hearing Loss

Social isolation is a major concern among people with hearing loss [1,2,3,4]. Uncorrected hearing loss can lead to both reduced interaction with others and to an increased subjective perception of loneliness [5,6,7]. Social isolation increases with increasing auditory threshold after hearing loss, and is also predicted by a rapid decline in understanding speech in noise [8,9]. However, social isolation is not an automatic consequence of hearing loss for everyone. The degree of hearing loss-associated social isolation may depend on sex and age, rural versus urban residence, education level, marital status, and use of assistive hearing devices [6,7,9,10,11,12,13,14]. Social isolation on its own is related to a constellation of negative outcomes for an individual’s overall health, including self-reports of physical and mental health [2,10,15,16], and the social isolation that accompanies hearing impairment also co-occurs with other health concerns, including depression, anxiety, and cognitive decline [2,10,15,16,17,18]. In spite of the strong correlation between hearing loss and social isolation, the neural mechanisms that might link these impairments have yet to be fully considered. In this synthesis and commentary, we will discuss the association between hearing loss and social isolation in humans and animal models, including the auditory plasticity independently induced by both factors. We will also consider the serotonergic system as a possible neural mechanism linking these two phenomena, because of its extensive connections with both the auditory system and brain areas that respond to and regulate social behavior.

The loss of input from the auditory periphery triggers a cascade of plasticity within the central auditory system, multiple aspects of which have been extensively reviewed. Following peripheral loss, the central auditory system undergoes a progressive rebalancing of excitation versus inhibition and synaptic plasticity that encompasses both ascending and descending systems (e.g., [19,20,21,22,23,24,25,26,27]). As a result of this rebalancing, the gain of local auditory potentials increases as auditory pathways ascend [28,29]. This type of rebalancing has been interpreted as an adaptive increase in central gain that compensates for decreased input from the periphery [30]. However, plastic changes that follow hearing loss also encompass spectral and temporal aspects of neural responses to acoustic stimuli (e.g., [31,32,33,34]) and have been associated with functional deficits that extend beyond higher perceptual thresholds. These deficits include difficulty in understanding speech, hypersensitivity to sound (hyperacusis), or perception of phantom sounds (tinnitus) [35,36,37,38,39,40,41,42].

Plasticity in the auditory system is triggered not only by hearing loss but also, surprisingly, by degree of social interaction. While this has been investigated to some extent in humans, most of this research has been conducted using animal models, especially songbirds. For example, socially isolated birds exhibit decreased selectivity of auditory-evoked activity in single cells [43,44,45,46], as well as changes at the population level, including decreased stimulus-specific activity [47] and an increased proportion of responsive sites [43,44]. Taken together, these studies suggest that a general effect of social isolation is to decrease response selectivity in the auditory system. Thus, it is possible that social isolation-induced changes in auditory processing could lead to altered perception of the acoustic environment. In fact, a few studies suggest that isolation impairs auditory perception. For example, socially deprived birds are unable to discriminate between songs of different frequencies or containing different song notes [48,49,50]. While it is unknown whether there is a causal relationship between social isolation and auditory plasticity in humans, social interaction plays an important role in supporting auditory function. Infants with experience interacting with a live person speaking Mandarin are better able to discriminate between phonetic units in Mandarin compared to infants who listened to either an audio or audio–video recording [51]. Given that social isolation is often concomitant with hearing loss, it is interesting that social isolation itself causes changes in auditory function. In an individual with hearing loss, the extent to which hearing loss and social isolation may act separately or synergistically to affect auditory function is unknown. It is therefore worthwhile to consider the mechanisms by which these two conditions might influence the auditory system.

Centralized neuromodulatory systems that respond to both hearing loss and social isolation and make contact with auditory areas could be involved in the auditory changes that accompany both conditions. Axons that release neuromodulators, including dopamine, acetylcholine, norepinephrine, and serotonin, are embedded within auditory pathways, but the neurons that synthesize these neurochemicals are often external to the auditory system and project to many other non-auditory brain regions [52]. At the level of auditory circuitry, neuromodulators regulate the balance of excitatory and inhibitory pathways through presynaptic and postsynaptic mechanisms [53,54,55,56,57]. As a result, neuromodulators alter the ways that auditory neurons respond to acoustic stimuli ([58,59,60]; reviewed in [52]) and gate information from different sources through key circuit elements like projection neurons [56,61]. Neuromodulatory neurons may receive afferent information from a wide range of integrative brain systems, and therefore, have the potential to provide ‘value-added’ feedback to the auditory system. This feedback may provide information on situational variables accompanying acoustic stimuli, such as social valence [62,63,64]. Although relatively little is known regarding the effects of hearing loss on neuromodulatory systems, these systems are highly sensitive to conditions such as social isolation [65,66,67].

The current paper addresses the convergence of hearing loss and social isolation on a single neuromodulatory system, the serotonergic system. This system has widespread projections throughout the brain and spinal cord, including within the auditory system [68,69,70]. Reflecting this broad distribution, serotonin is involved in regulating multiple kinds of social behaviors, mood and affect, sensory and motor processing, and the connectivity within and among neural circuits [71,72,73,74,75,76]. Hearing loss and social isolation show parallels in their influence on serotonergic pathways. Each condition affects serotonergic pathways within the auditory system. Each factor also acts at multiple mechanistic levels, incorporating effects that are both presynaptic and postsynaptic to serotonin release. The following sections illustrate these points by briefly describing the working relationship between the serotonergic and auditory systems, and by reviewing the effects of induced hearing loss and social isolation on these interacting systems.

## 2. Defining Serotonin–Auditory Interactions

Serotonergic terminals are observed from the cochlea through auditory cortical regions and the manipulation of serotonin strongly influences auditory neurons both in vivo and in vitro. An interdisciplinary concept of the function of neuromodulators like serotonin is that they reconfigure excitatory and inhibitory circuitry [77,78,79]. This concept is a good match for the plasticity caused by serotonin in auditory circuits. This section briefly outlines a framework of circuit reconfiguration by serotonin within the auditory system, a topic that has also been reviewed elsewhere [52,64].

**Figure 1:**
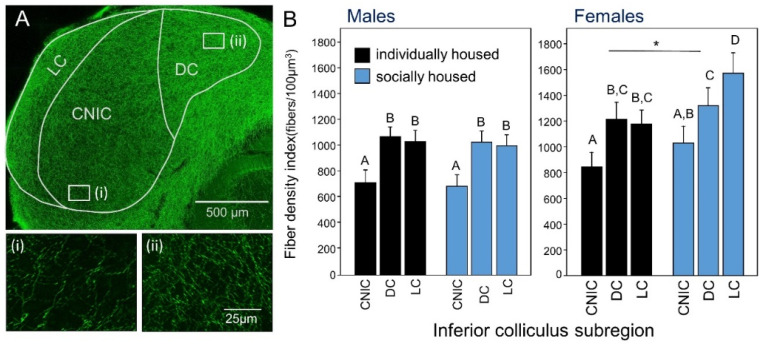
(**A**) Axons from serotonergic neurons form a dense network in the inferior colliculus (IC). Photomicrographs with higher magnification labeled (i) and (ii) at the bottom of panel A correspond to the white boxes in the top panel. The subdivisions of the IC are also indicated on this photomicrograph: CNIC = central nucleus of the IC; DC = dorsal cortex of the IC; LC = lateral cortex of the IC. (**B**) Fiber densities in mice housed individually versus in social groups for one month after weaning, across distinct subregions of the IC. Fiber densities are highest in females housed socially (* *p* < 0.05). Figures adapted from reference [88] with permission from the authors.

Networks of serotonergic fibers in auditory regions express regular varicosities (presumed release sites) that show *en passant* morphology, with release sites that may be closely apposed to cell bodies [70], or even cluster around cell bodies in basket-like formations [80]. The density of serotonergic fibers varies across auditory regions and within functionally distinct subregions of auditory nuclei (Figure 1A); some aspects of these patterns are species-specific [52]. Tract tracing studies have identified the major source of serotonergic input to auditory regions as the dorsal raphe nucleus (DRN; cell groups B6 and B7), although smaller contributions originate in other raphe nuclei [68,81,82]. Multiple subgroups of DRN neurons project to at least some regions of the auditory system. These subgroups respond differently to contexts such as physical stress and social interaction, or are associated with social behaviors like aggression [81]. The pathways from the DRN to the auditory system could therefore potentially convey information on salient events. Following release, serotonin acts through receptors expressed by auditory neurons. Members of many of the seven major families of the serotonin receptor have been documented in auditory regions through ligand binding, immunolabeling, quantitative PCR (polymerase chain reaction), and via the effects of selective serotonin receptor agonists and antagonists (e.g., [57,83,84,85,86,87]).

Within this anatomical framework, serotonergic function within the auditory system has been assessed by directly measuring serotonin availability during different kinds of events, and by manipulating serotonin or its receptors, and subsequently, monitoring neural or behavioral responses. The direct measurement of serotonin in awake and behaving rodents has shown that serotonin increases in response to both acoustic and non-acoustic stimuli. The presentation of white noise increases serotonergic activity in auditory regions caudal to the thalamus [89,90]. Both the acoustic stressor of noise and the non-acoustic stressor of spatial confinement are capable of elevating serotonergic activity [89,91]. On the other hand, some stressors with strong behavioral effects in rodents, like exposure to a chemical component of fox urine, do not alter serotonergic activity [89]. These findings suggest that serotonin release in the auditory system is somewhat selective within broad categories of behavioral events like ‘stressors’.

In the auditory midbrain, serotonergic activity is also elevated during multiple types of social interaction: in males with either male or female social partners, and in females with male social partners [62,63,92]. In all of these different types of interactions, interindividual variation in serotonergic activity parallels variation in behavior. However, serotonergic activity in the auditory midbrain does not simply reflect the acoustic social environment, in that serotonin does not positively correlate with increased vocal behavior. In fact, serotonin may correlate negatively with vocal behavior. For example, in males and females interacting with male social partners, serotonergic activity is highest for subjects that show the most locomotor activity and social investigation, indicating increased overall behavioral arousal [62,92]. In contrast, in males interacting with females, serotonergic activity negatively corresponds to the behavior of the female social partners [63]. When females make a larger number of broadband vocalizations, often interpreted as an indication of female rejection in mice, male serotonin does not increase. When females show less rejection of males, serotonergic increases are larger. Across multiple types of behavioral interactions, serotonin therefore reflects the salience of a social interaction for individual mice, as reported by the vocal and non-vocal behaviors of mice or their social partners.

The responsiveness of auditory neurons to fluctuations in serotonin has been assessed by bypassing endogenous patterns of serotonin release and directly manipulating serotonin or specific types of serotonin receptors within the auditory system. These kinds of manipulations have effects that are observed at the level of individual neurons, specific neural circuits, and neuron populations. The effects of these manipulations depend on the cell type being monitored and the serotonergic receptor type that has been activated or blocked. For example, in the inferior colliculus (IC), 5-HT2A receptors facilitate inhibitory transmission by presynaptically increasing the frequency of spontaneous inhibitory postsynaptic events [93]. Serotonin receptors may even have effects that are specialized for specific subcellular compartments such as the axon initial segment [55]. The effects of serotonin at the level of single neurons converge into coherent effects on the function of specific auditory circuits. In the dorsal cochlear nucleus (DCN), serotonin acts on both excitatory and inhibitory neurons through different receptor types that converge at the level of fusiform cells, which project to other auditory nuclei [56]. The net result is to strengthen pathways that carry non-auditory information and to dampen pathways that carry auditory information, reconfiguring the output of the DCN.

At the level of the neural population in the IC, manipulating serotonin availability alters the number of neurons expressing the activity-dependent immediate early gene product cFos in female mice [94]. The effect of serotonergic manipulation depends on whether mice have undergone direct social interaction or simply listened to the playback of calls. The effect of serotonin further depends on the females’ estrous states (corresponding to ovarian cycles and behavioral receptivity), and so depends on internal state as well as the external context. Reflecting another type of population-level effect of serotonergic manipulation, auditory brainstem responses (ABRs) are also influenced by serotonin depletion, and by selective receptor agonists and antagonists [95]. Both the amplitudes and latencies of multiple ABR waves show effects that depend on the receptor type being manipulated. Overall, effects of serotonergic manipulation are more often seen when ABRs are evoked by low-frequency tones than by high-frequency tones, suggesting that the effects of serotonin access the basic tonotopic organization of the auditory system.

Given these effects of serotonin within many regions of the auditory system, it is hardly surprising that serotonin can influence auditory processing and acoustic perception in human subjects. High levels of serotonin have been associated with a shallower slope of Wave V and the N1/P2 potential of auditory-evoked responses [96,97,98], and a polymorphism of the serotonin transporter is associated with an improved signal-to-noise ratio of the frequency following response [99]. Some aspects of speech perception, including filtered word perception and the perception of target sentences in competing sound, are improved in patients on selective serotonin reuptake inhibitors (SSRIs) compared to the same patients in an unmedicated condition [97,100]. On the other hand, some studies have found few effects of SSRIs on multiple measures of auditory perception (e.g., [101]). Likewise, although there is some evidence for an effect of SSRIs on the perception of tinnitus, these effects are extremely mixed, with different studies showing a reduction in tinnitus, an increase in tinnitus, or no effect [102,103,104]. Together, all of these studies suggest that the influence of serotonergic pathways on auditory perception in human subjects might be highly condition-dependent.

**Summary**

Presynaptic and postsynaptic elements of serotonergic pathways are present throughout the auditory system. Serotonin responds to broad classes of events such as stressful events or social interaction. Within the category of social interaction, serotonergic release parallels the salience of specific interactions. Once released, serotonin has cell-specific and receptor-specific effects that functionally reconfigure auditory circuits. Table 1 summarizes selected articles addressing the functional effects of serotonin at a wide range of sites within the auditory system. Overall, serotonin induces plasticity in the auditory system that is evident at multiple organizational levels.

## 3. Plasticity in the Serotonergic System

Given the scale of serotonergic innervation of the auditory system and the ability of serotonin to induce plasticity in auditory processing, it is striking that the serotonergic system itself exhibits extensive plasticity. Plasticity can result from long-term changes in acoustic input, but also occurs in response to changes in the general social environment. This type of sensitivity to environmental influence can serve an important function in adapting serotonergic regulation to long-term changes in internal and external conditions. Plasticity in serotonin–auditory interactions is expressed at both presynaptic and postsynaptic mechanistic levels. That is, changes occur in both the serotonergic fibers and the dynamics of serotonin release, and in the expression of serotonin receptors by auditory neurons.

## 4. Hearing Loss and the Serotonergic System

Sound-induced hearing loss alters the densities of serotonergic fibers and the functional dynamics of serotonin release and reuptake. Following monaural exposure of mice to an 8 kHz tone at 113 dB, axons that are immunopositive for the serotonin transporter (SERT+) in the inferior colliculus (IC) are altered in density [119]. Serotonergic axons are relatively less dense in the IC contralateral to the acoustically traumatized ear, and relatively more dense in the IC ipsilateral to the protected ear. These changes occur equally in the subregions of the IC receiving more descending and non-auditory input (the lateral and dorsal cortices) versus the central subregion, which receives a greater proportion of ascending auditory information. One of the most interesting aspects of this finding is the lateralization of the effects of acoustic trauma, which suggest the possibility that a decrease in SERT+ fiber density occurs as a result of local interaction of the SERT+ fibers with neurons experiencing regionally specific plasticity or damage [120].

Following bilateral tonal acoustic trauma of rats, densities of SERT+ axons are also lower in multiple auditory regions, including the cochlear nucleus, IC, and auditory cortex relative to a non-trauma group [120]. In parallel, the uptake of a radioactive SERT ligand is reduced in sound-exposed rats relative to controls. Notably, the effects of sound exposure are equally strong in many non-auditory regions, including the hypothalamus, hippocampus, striatum, and frontal cortex. Exposure to acoustically traumatizing sound therefore results in broad changes in the expression of SERT.

Postsynaptic changes in serotonergic pathways are also altered by hearing loss. Following hearing loss induced by bilateral exposure to a 10 kHz tone at 116 dB, the expression of multiple types of serotonin receptor are altered in the IC. Receptors in the 5-HT1 family, such as the 5-HT1A and 5-HT1B receptors, are strongly expressed in the IC [83,84,85]. Acoustic trauma leads to an approximately threefold increase in expression of the Htr1A gene (5-HT1A receptor) and a tenfold increase in expression of the Htr1B gene (5-HT1B receptor), relative to sham treatment [85]. Across sham and experimental groups, the degree of hearing loss correlates positively with the level of Htr1B expression. In contrast, no significant changes occur for genes encoding the 5-HT2A or 5-HT3A receptors. In contrast to non-auditory regions showing reduced SERT after hearing loss, no changes in serotonin receptor gene expression are observed in a non-auditory brain region innervated by the DRN, the hippocampus.

Speculation about the functional consequences of these changes in gene expression can be supported by the distinct roles of these receptor types. The 5-HT1A receptor is often expressed somatodendritically or in the axon initial segment, and often suppresses neural firing [55,59,121]. In the IC, activation of this receptor decreases spontaneous and sound-evoked spikes, and delays first-spike latencies [59]. Increased expression of this receptor type could therefore create heightened suppression of acoustically evoked activity in a subset of neurons during serotonin release. In contrast, the 5-HT1B receptor is expressed near the presynaptic terminal, where it decreases transmitter release [53,122]. The expression of this receptor in the IC therefore captures a likely suppressive effect on the synaptic outputs of IC neurons. Across both of these receptor types, hearing loss could lead to heightened regulation of neural responses to sound during serotonin release.

Changes in the expression of 5-HT receptors are also observed after hearing loss associated with age or following cochlear ablation [86,87]. The 5-HT2B receptor is upregulated in the cochleae of old mice relative to young mice, regardless of the severity of hearing loss. In the IC, however, the 5-HT2B receptor is expressed more strongly in old mice with severe hearing loss than in old mice with mild hearing loss. Across individuals, the amount of 5-HT5B expression correlates with the amplitudes of DPOAEs, and with ABR thresholds, particularly ABRs in response to high-frequency tones. Finally, cochlear ablation causes a temporary decrease in expression of the 5-HT5B receptor (3 and 21 d, baseline by 90 d), and a long-term increase in expression of the 5-HT2C receptor over 90 days. These receptor types have been less well explored in terms of their function in auditory regions, but the 5-HT2B receptor has been associated with the promotion of plasticity related to mechanisms including calcium regulation and inflammation (discussed in reference [86]).

Some of these effects of exposure to loud sound on the serotonergic system could occur in part through the activation of stress-responsive physiological systems rather than through a primary effect on the auditory system. Noise exposure itself is a stressful event that can increase serum glucocorticoid levels and alter the expression of glucocorticoid receptors, although these effects are dependent on the type of stressor and brain region [123,124]. Three classes of findings suggest that stressors associated with hearing loss independently affect serotonin–auditory interactions. The first type of finding is that serotonergic activity increases during exposure to noise, as described in an earlier section. Second, sham treatments that involve anesthesia or surgery alter the expression and function of serotonin receptors. In the IC, sham treatment including anesthesia and the measurement of ABRs causes a decrease in the expression of Htr1B mRNA [85]. In the auditory cortex, a sham cochlear ablation results in functional changes in the response to serotonin that may occur through multiple types of serotonin receptors [125]. These kinds of findings are consistent with studies showing that the serotonergic system is sensitive to a range of stressors [126,127], and illustrate the importance of sham control groups. A third highly specific finding is that a substance that reduces oxidative stress, resveratrol, can protect SERT-immunoreactive fibers in both auditory and non-auditory regions from a decline following tonally evoked hearing loss [120]. Thus, blocking specific physiological components of a stress response can prevent noise-induced damage to the serotonergic system. Together, these considerations suggest that the stress accompanying some kinds of hearing loss may contribute to plasticity in serotonergic pathways both inside and outside of the auditory system.

**Summary**

Hearing loss is associated with presynaptic and postsynaptic changes in the serotonergic system within auditory regions, but some of these changes also occur in other brain regions. Stressors associated with the induction of hearing loss may independently contribute to the plasticity in local serotonergic pathways.

## 5. Social Isolation and the Auditory System

As a factor on its own, social isolation has significant consequences for auditory function. While some work has demonstrated an effect of social interaction on auditory responses in humans (e.g., [51]), the bulk of this research has been conducted using animal models, especially birds. Songbirds are of particular interest, because vocal communication in songbirds is learned and social contact can be easily manipulated [128]. Social deprivation influences auditory processing at multiple functional levels.

At the level of single cells, the response properties of individual auditory neurons change in ways that suggest that auditory processing is more finely tuned in socially housed birds. Auditory neurons of socially housed birds show less noisy, more precise responses to sound compared to isolated birds [43,44]. Furthermore, auditory neurons respond more selectively to vocalizations in social birds compared to isolated birds [44]. Social isolation also impairs discrimination and selectivity of single neurons to different classes of song in an auditory area involved in assigning meaning to auditory stimuli, the caudomedial nidopallium (NCM) [45,46]. Taken together, these studies suggest that a main effect of social isolation is to decrease the precision and selectivity of evoked activity in single auditory neurons.

Social isolation also alters the neural processing of sound on a larger scale. For example, when compared to socially housed birds, birds raised in isolation have more responsive sites across an avian auditory forebrain area, Field L [43,44]. This finding is especially significant, given that adult songs were broadcast to birds in both the social and isolated housing conditions. Thus, the major difference between the isolated and group-housed treatments was the social setting, not the presence or absence of song [43,44], suggesting that social stimulation itself (not merely acoustic environment) is an important factor that affects auditory function. Functional magnetic resonance imaging in the same auditory area, Field L, reveals that birds with a greater degree of social enrichment have the strongest stimulus-specific activity, whereas completely isolated birds do not show any selectivity [47]. Collectively, these studies suggest that social engagement is a major factor contributing to the response properties of the auditory system. The opposite pattern emerges in different areas of the avian auditory forebrain when measured with immediate-early gene (IEG) expression. In both the caudomedial mesopallium and the caudomedial nidopallium, isolated birds showed decreased IEG activity in response to conspecific calls compared to socially housed birds [129]. However, the IEG measured in this study, ZENK (the avian homologue of genes zif268, EGR-1, NGFI-A, and krox24), may not be a direct correlate of neural activity but instead may be an indicator of neural plasticity, since the ZENK response in the NCM is correlated with the strength of song learning [130,131,132], and song learning is impaired by suppression of the cellular pathway that induces the ZENK response [133]. Taken together, these studies demonstrate that social isolation dramatically changes auditory processing at functional levels above that of single auditory cells. These isolation-induced changes in auditory function could lead to changes at the perceptual level.

In fact, a few studies suggest that social isolation also shapes auditory perception. For example, completely socially isolated birds of several species are unable to discriminate between songs based on frequency; socially deprived birds are also unable to discriminate between different song notes [48,49,50]. Interestingly, some of the loss in discrimination ability can be rescued by increasing the amount of socialization. Birds raised with siblings (but still without adult contact) show deficits in absolute frequency discrimination, but are able to discriminate songs based on relative frequency and based on different song notes [49]. This suggests that not only does social isolation itself affect auditory perception, but the degree of social isolation affects the degree of perceptual impairment.

While deprivation of social stimulation alters auditory responses on their own, social deprivation also affects the integration of non-auditory sensory input with auditory processing. In birds raised with adults, images of familiar versus unfamiliar birds differently alter auditory-evoked activity [134]. In contrast, birds reared without adult experience show no difference in cross-contextual modulation of auditory processing depending on familiarity of the visual stimulus. In other words, birds raised in isolation from adults lack a multisensory representation of familiarity [134]. These findings raise the possibility that social deprivation influences how extra-auditory information is conveyed to the auditory system.

Research in songbirds suggests that social isolation itself influences auditory processing, but whether this phenomenon extends to other taxa, such as mammals, is relatively underexplored. However, there is a strong body of literature in mammals, particularly in laboratory rodents, demonstrating that social deprivation causes dramatic effects on behavior [65,67,135], and some of the isolation-induced changes in behavior suggest that there may be underlying alterations in auditory processing. For example, social isolation alters the prepulse inhibition of the acoustic startle response in rodents, with isolated rats and mice generally showing an attenuated effect of prepulse stimuli compared to group-housed animals [136,137,138,139,140,141,142]. While most of this work has been conducted in males, there is evidence to suggest that the effect of prepulse inhibition is also attenuated in isolated females [143]. Interestingly, when the effects of social isolation in both sexes are investigated within the same study, these studies report a sex difference, with males being especially susceptible to isolation-induced deficits in both baseline acoustic startle [144] and prepulse inhibition of acoustic startle [145]. These findings suggest that sex is another important factor that may interact with social isolation and sensorimotor processing.

In addition to the research on the acoustic startle response, one study has explored whether social experience shapes auditory perception in mammals, by investigating the influence of social versus individual housing on auditory discrimination in mice [146]. Housing-dependent differences in auditory discrimination of mouse vocalizations, as assessed by an operant conditioning task, were subtle, but still suggest that social isolation may alter auditory perception. Although mice from both housing conditions showed similar degrees of discrimination ability, isolated mice used different spectrotemporal parameters to differentiate between acoustic stimuli when compared to their socially housed counterparts [146]. Taken together, behavioral responses to sound do seem to be altered by social deprivation, raising the possibility that there may be changes in auditory processing induced by isolation in mammals. However, it cannot be ruled out that the isolation-induced effects on acoustic responses described here could be elsewhere in the pathway between sensory reception and motor output. For example, the effects of social isolation on prepulse inhibition of the acoustic startle response may in part be due to neurochemical changes in the nucleus accumbens [136]. Thus, more research is needed to determine the extent to which social isolation of mammals has an effect on auditory responses, at multiple functional levels.

**Summary**

Similar to hearing loss, social isolation induces plasticity in auditory processing and may influence the integration of acoustic and non-acoustic information within the auditory system. The majority of this work has been conducted using songbirds as a model system. Future studies should investigate whether social deprivation of mammals causes similar deficits in auditory processing.

## 6. Social Isolation Influences Serotonin in the Auditory System

The serotonergic system provides a source of non-acoustic input to the auditory system and is sensitive to the social environment; it is therefore one potential pathway through which social isolation could influence auditory function. Whether social isolation reconfigures this pathway is just beginning to be explored. Two studies [88,147] suggest that social impoverishment modifies serotonergic anatomy and function in the auditory system at the level of the midbrain—the IC. In both studies, the effects of social isolation were investigated by manipulating the chronic social housing conditions of postweaning mice, a manipulation with behavioral and physiological consequences that include effects on the serotonergic system in non-auditory regions of the brain (see below). Long-term social isolation influences SERT+ fiber density in the IC in a sex-dependent manner, with an effect of social isolation demonstrated by females but not males [88] (Figure 1B). Individually housed female mice differed in SERT+ fiber density from their socially housed counterparts in two ways. First, individually housed females showed an overall lower SERT+ fiber density compared to socially housed females. Second, individually housed females also showed a different distribution of SERT+ fibers across subregions within the IC. While females in both housing conditions showed higher densities of SERT+ fibers in the cortex of the IC compared to the central nucleus of the IC, there was no difference in SERT+ fiber density between the dorsal and lateral cortices of individually housed females. In contrast, socially housed females demonstrated a higher density of SERT+ fibers in the lateral cortex compared to the dorsal cortex of the IC (Figure 1B). These findings are interesting given that the lateral cortex of the IC receives ascending and descending auditory input, as well as input from non-auditory sensory areas [22]. Thus, altered serotonergic innervation of the lateral cortex in socially deprived females may not only influence serotonergic modulation of auditory responses, but also affect how extra-auditory input influences auditory processing, via serotonergic modulation of those pathways.

In the Keesom et al. [88] study described above, the sex-dependent effect of social isolation on SERT+ fiber density was paralleled by a sex-specific effect of social isolation on body weight. Socially isolated female mice showed decreased body weight compared to socially housed females, whereas there was no difference in body weight in males depending on social housing conditions. This finding is comparable to a previous report of decreased weights in females, but not males, due to social deprivation [148]. Given that reduced body weight is one indicator of stress in rodents [149], these findings suggest that the effects of isolation on SERT+ fibers in the auditory system may be tied to psychosocial stress.

**Figure 2:**
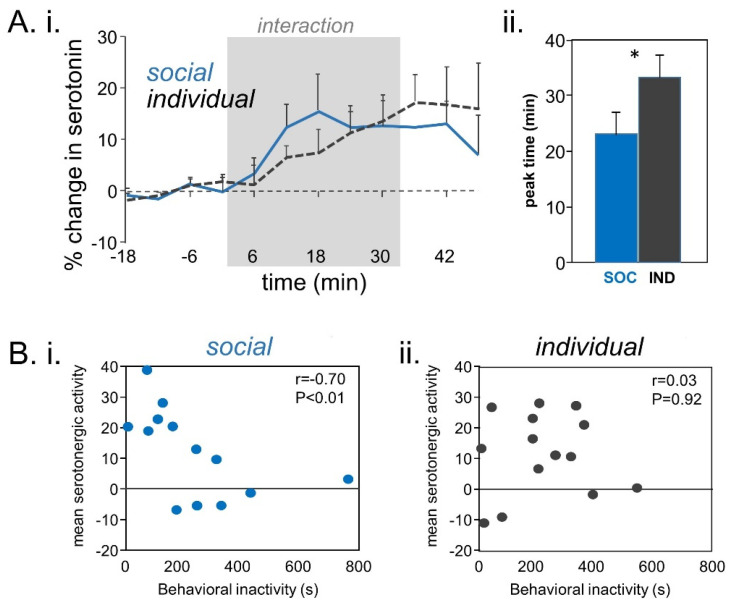
(**A**) (**i**) Changes in voltammetrically measured serotonin during male–male interactions in mice. Serotonergic increases occur in males interacting with novel social partners regardless of whether males were housed in groups or individually after weaning. (**ii**) However, time to the peak change in serotonin is longer in individually housed males (* *p* < 0.05). (**B**) (**i**) Across individual mice housed in groups, the integrated increase in serotonin inversely correlates with the overall level of activity. (**ii**) No correlation between serotonin and inactivity is seen for individually housed males. Adapted from reference [147] with permission from the authors.

Although there is no effect of social isolation in males on the density of SERT+ projections to the IC, depriving male mice of social contact does alter directly measured serotonergic activity in the IC triggered by the presence of a social partner [147]. In this study, despite demonstrating differences in the mean durations of social investigatory behavior and behavioral activity, socially isolated and social housed adult male mice did not differ in mean amplitudes in serotonergic activity during social interaction. Instead, the chronic social environment influenced the dynamics of serotonergic availability in the IC. Isolated males exhibited a slower rise in serotonergic activity compared to socially housed males, with an increased latency to elevated serotonergic activity above baseline levels and increased time until maximal serotonergic activity (Figure 2A). Not only did social isolation influence the timing of the serotonergic response to a social partner, but it also affected the relationship between serotonergic activity in the IC and behaviors displayed during the social encounter. For example, there was a negative relationship between overall serotonergic activity and behavioral inactivity across interactions between socially housed males, which replicated a finding from a previous study [62,147] (Figure 2B). Socially housed males also showed a positive correlation between serotonergic activity and social investigation behavior. On the other hand, long-term social isolation disrupted these serotonin–behavior relationships: there was no relationship between serotonin and behavioral inactivity, or between serotonin and social investigation, in socially isolated males. These findings suggest that social isolation may interfere with the serotonergic system’s ability to convey contextual information to the auditory system. Furthermore, because serotonin influences auditory responses, isolation-induced changes in socially triggered serotonergic activity may lead to context-dependent effects of social isolation on auditory processing. Future studies should investigate this possibility.

**Summary**

Social isolation influences the interaction between the serotonergic and auditory systems, with effects on serotonergic fiber density and serotonergic activity in the auditory midbrain that match changes in other factors sensitive to isolation, including body weight and behavior.

## 7. Hearing Loss and Social Isolation have Extra-Auditory Effects

Although this synthesis paper has focused on the auditory system, hearing loss and social isolation both influence the serotonergic system outside of auditory regions. This has been best explored for social isolation. Social isolation influences serotonergic axons, activity, and metabolism throughout the brain, including regions involved in cognition, memory, emotion, and reward. In comparison to the sex-specific decrease in serotonergic fiber density that it causes in the auditory system, social isolation has mixed effects on serotonergic axons across several extra-auditory brain regions. Isolation decreases serotonergic fiber density in the hippocampus [150] but increases fiber density in the dorsal caudate-putamen [151] and prefrontal cortex [152]. Serotonergic fiber density in the amygdala is also sensitive to social isolation; however, different studies have reported opposite directions of the effect of isolation [151,153]. Along with its effects on serotonergic axon density, social isolation influences serotonergic availability and dynamics in non-auditory brain areas, with the direction of the effect depending on the brain region and the nature of the evoking stimulus. Socially deprived animals show increased serotonergic activity in the prefrontal cortex [154] and hypothalamus [155] in response to a social partner, and in the nucleus accumbens in response to aversive stimuli [156,157]. In contrast, social isolation leads to decreased serotonin release in the prefrontal cortex in response to aversive stimuli and non-social reward [158,159], as well as in the hippocampus in response to a novel environment [160,161]. Not only is the release of serotonin influenced by social deprivation, but the metabolism of serotonin to its metabolite, 5-HIAA (5-hydroxyindoleacetic acid), is also affected [162,163,164,165]. The effect of induced hearing loss on the serotonergic system outside of auditory regions has been less well explored, but hearing loss decreases serotonergic fibers in some extra-auditory brain regions, including the hypothalamus, striatum, and frontal cortex [120,166].

The extra-auditory effects of both hearing loss and social isolation are particularly interesting in light of the important role of interaction among auditory and non-auditory regions in giving rise to perceptual dysfunction following hearing loss. For example, relative to human subjects with equivalent hearing loss, subjects with tinnitus have anatomical and functional differences in brain regions, including the ventromedial prefrontal cortex and the amygdala [167,168,169,170]. These findings have led to a model of descending control from these non-auditory regions to the thalamus, which gate a tinnitus signal originating in abnormal activity within the auditory system [169]. Other imaging studies in human subjects have shown that sensorineural hearing loss is associated with a widespread decrease in connectivity in multiple functional brain networks involved in decoding or assessing the meaning of the acoustic environment. These networks include auditory cortical regions and the insula, amygdala, and different cerebellar subregions that combine multimodal sensory information with information on internal state [171,172,173,174]. For some networks, the lack of connectivity further correlates with measures of speech comprehension, as well as general measures of cognitive performance or affective state.

The interactive effects of hearing loss and emotional systems on perception are also illustrated by a three-way interaction between hearing loss, the subjective perception of social isolation, and the affective perception of acoustic stimuli. Subjects with hearing loss show dampened emotional ratings of affectively charged sounds, use a smaller range of values to describe such sounds, or take longer to respond to affective sounds but not neutral sounds in comparison to normal hearing listeners [175,176,177,178]. The degree of hearing loss may further correlate with depression and anxiety [177]. In one study, both hearing loss and the perception of social disconnectedness were related to emotional ratings of a test battery of non-speech sounds [178]. These findings show that long-term accommodation to hearing loss by the brain incorporates neural systems that regulate cognitive and affective processes, which may be involved in some of the devastating perceptual dysfunctions associated with hearing loss.

**Summary**

Hearing loss and especially social isolation influence presynaptic and postsynaptic elements of the serotonergic system outside of the central auditory system, including in brain areas related to memory, reward, and emotion. This is particularly interesting given that alterations in the functional connectivity between auditory and non-auditory regions are associated with deficits in the perception of acoustic stimuli, and that the perception of social isolation co-occurs with an altered emotional response to sound.

## 8. Do Hearing Loss and Social Isolation Physiologically Converge?

Although the current paper presents evidence that hearing loss and social isolation each influence the serotonergic system, whether their effects actually converge on the same targets, and if so, at what levels of organization, have not yet been experimentally explored. For example, multiple subgroups of serotonergic neurons with different responsiveness to external events may innervate the same auditory regions [81,82]. It is therefore possible that, although hearing loss and social isolation both decrease serotonergic axon density, they do so by targeting functionally distinct axons. Likewise, although both social isolation and hearing loss change the expression of serotonin receptors either within or outside of the auditory system, it is not clear whether they influence the same receptor types within the same auditory regions.

These uncertainties allow for the possibility of significant nonlinearity in the interactions between hearing loss and social isolation at the level of auditory processing and perception. Possible types of interaction include synergy, in which the effects of hearing loss and social isolation are greater than either alone. In this model, multiple separate ‘hits’ to the serotonergic system caused by hearing loss and social isolation, could create extreme dysregulation of auditory processing. Conversely, maintained social connection could minimize negative effects of hearing loss on serotonin–auditory interaction. This could fall within the ‘social buffering’ hypothesis, in which social contact counteracts some of the negative effects of stressors [179]. In contrast to these scenarios, different levels or types of stressor could have protective effects on the serotonergic system, as glucocorticoids do at the auditory periphery [180,181]. Which types of interaction are observed could further depend on the underlying physiological state or genetic factors that regulate the serotonergic system [182,183].

On a broader scale, the possibly convergent effects of hearing loss and social isolation on the serotonergic system play out within the context of many interacting sensory and cognitive systems that may themselves be sensitive to these factors. A useful framework for envisioning different types of interactions among auditory and non-auditory systems is the increasingly supported link between hearing loss and cognitive decline in older adults. Difficulty in hearing is predictive of cognitive deficits in a range of tasks assessing memory, executive function, and speed of response [184,185,186,187,188,189,190]. Several types of non-exclusive explanations may account for this link [189,191,192,193,194]. In addition to a ‘common cause’ model, in which a neurodegenerative process targets both sensory and cognitive systems, hearing loss may be causal to cognitive decline in several ways. The ‘cognitive load’ hypothesis posits that perceiving important signals like speech in the face of hearing loss forces the use of other sources of information like contextual cues. Such ‘effortful listening’ presents a burden to finite cognitive resources that decreases performance in other tasks [195,196,197]. The ‘deprivation hypothesis’ supposes that decreased input from the sensory periphery causes central plasticity that results in the ultimate degeneration of not only auditory but cognitive neural pathways [198,199].

The idea that social isolation itself may play an important role in cognitive decline following hearing loss is supported by a recent study modeling the causal interactions of hearing loss, social isolation, and subjective loneliness in cognitive outcomes [200]. The study’s authors concluded that social isolation mediated some, although not all, of the effects of hearing loss on a decrease in episodic memory. These kinds of complex interactions underline the importance of exploring neural systems, like the serotonergic system, that can integrate information on different types of peripheral or environmental changes, and co-regulate sensory and cognitive systems. The serotonergic system is not the only neurochemical system that can potentially play this role, however. Multiple centralized neuromodulatory systems widely innervate sensory and integrative brain regions, and respond to changes in environmental conditions [64]. For example, noradrenergic neurons within the locus coeruleus are sensitive to behaviorally important information, such as social context or stress, are affected by social experience, influence connectivity in neural networks related to salience and affect, and alter performance on sensory and cognitive tasks [201,202,203,204,205,206]. These kinds of similarities in some of the aspects of how serotonergic, noradrenergic, and other neurochemical systems work have led to the view that these systems may cooperate in regulating integrative neural functions [74,207].

**Figure 3:**
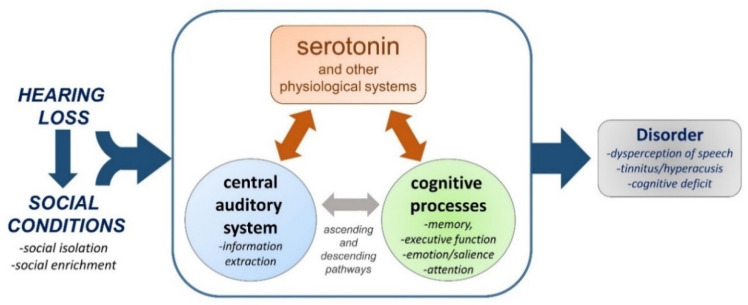
Conceptual diagram of the serotonergic system as a regulator of interacting neural systems that are affected by hearing loss and social isolation. Plasticity in the regulation of interacting auditory and extra-auditory circuitry by serotonin could lead to consequences for perceptual and affective auditory responses to sound.

Models of the links among hearing loss, isolation, and perceptual or cognitive outcomes that incorporate physiological systems can serve an important role in illustrating the potential for ameliorating some of the consequences of hearing loss and social isolation. For example, some of the hypotheses linking hearing loss with cognitive decline predict that assistive hearing devices should prevent or reduce cognitive deficits. There is increasing evidence that such sensory interventions do decrease cognitive declines, or slow the rate of decline (e.g., [208,209,210]). Likewise, the model depicted in Figure 3 suggests that pharmacological agents targeting the serotonergic system could alleviate the negative consequences of hearing loss and social isolation. Some evidence supports this possibility. For example, administration of the SSRI citalopram reverses the decline in serotonergic fibers following hearing loss both in auditory and non-auditory regions [120]. This drug similarly reverses both neural and cognitive deficits associated with social isolation [211]. On the other hand, the use of SSRIs to counter the perception of tinnitus has been met with mixed results [104,212,213,214].

## 9. Concluding Thoughts

Social animals such as rodents, songbirds, and humans may have the opportunity to adapt their behaviors to a wide range of social environments, from enriched to impoverished networks of social contacts. This capability may involve neuromodulatory systems, such as the serotonergic system, given that serotonergic pathways respond to sensory stimuli and external events and are also highly attuned to the social environment. These functions fit the description of a system that could respond to comorbid conditions such as hearing loss and social isolation. Disruptions in neuromodulatory function due to hearing loss and/or social isolation may also have consequences related to the role of these same neurochemical systems in regulating intrinsic neural processing in many brain regions, as well as in regulating connectivity among interacting networks. These dual roles make these systems, including the serotonergic system, a potentially valuable key for understanding the origins of links among social environment, sensory function, communicative dysfunction, and cognition.

## Figures and Tables

**Table 1:** Selected articles describing some of the functional effects of serotonin across a range of auditory sites. Abbreviations: DCN = dorsal cochlear nucleus; AVCN = anteroventral cochlear nucleus; PVCN = posteroventral cochlear nucleus; MNTB = medial nucleus of the trapezoid body; LSO = lateral superior olive; IC = inferior colliculus; MGB = medial geniculate body.

Region	Reference	Receptor Type	Proposed Function of Serotonin
Cochlea	[105]	n/a	synaptically released
DCN	[56,61]	multiple	enhances multimodal pathways while dampening auditory pathways
	[106]	5-HT2, other	enhances excitability (5-HT2), decreases excitability (other)
PVCN, AVCN, and DCN	[107]	likely multiple	inhibits and facilitates sound-evoked spiking
MNTB	[53]	5-HT1B	presynaptically decreases glutamate release, developmentally regulated
LSO	[108]	5-HT1, 5-HT2	suppresses evoked excitatory postsynaptic currents, induces spontaneous inhibitory postsynaptic currents, developmentally regulated
	[109]	n/a	promotes development of projections from LSO to IC
IC	[110]	5-HT1A, 5HT1B	5-HT1A suppresses sound-evoked spiking, 5-HT1B increases sound-evoked spiking via GABAergic suppression
	[93]	5-HT2A	enhances spontaneous inhibitory postsynaptic potentials
	[111]	5-HT3A	activity-dependent response gain adjustment
	[112]	5-HT3A	response gain adjustment
	[94]	n/a	context-dependent alteration of immediate early gene expression
MGB	[113]	n/a	reduces burst firing
Cortex	[114,115]	5-HT1A, 5-HT2	reduces excitatory and inhibitory postsynaptic currents
	[116]	5-HT3A	excites inhibitory interneurons that regulate critical period timing
	[117]	5-HT2, 5-HT3	regulates synaptic plasticity
	[118]	5-HT2	regulates plasticity in frequency tuning
